# ANTIHYPERTENSIVE DEPRESCRIBING AND DISABILITY IN VA NURSING HOME RESIDENTS WITH AND WITHOUT DEMENTIA

**DOI:** 10.1093/geroni/igad104.2283

**Published:** 2023-12-21

**Authors:** Xiaojuan Liu, Laura Graham, Bocheng Jing, Chintan Dave, Yongmei Li, Kathy Fung, Michelle Odden

**Affiliations:** Stanford University, Stanford, California, United States; VA Palo Alto Health Care System, Palo Alto, California, United States; NCIRE, San Francisco VA Medical Center, UCSF, San Francisco, California, United States; Rutgers University, New Brunswick, New Jersey, United States; Stanford University, Stanford, California, United States; San Francisco VA Medical Center, San Francisco, California, United States; Stanford University, Stanford, California, United States

## Abstract

Deprescribing antihypertensives is of growing interest in geriatric medicine, yet clinical trial evidence is lacking. We leveraged the Veterans Affairs (VA) electronic health data and emulated a target trial of antihypertensive deprescribing on longitudinal change in disability, measured by activities of daily living (ADL), ranging 0-28. Residents included were ≥65 years with long-term stays ≥12 weeks from 2006-2019 (n=42537). We excluded those not on antihypertensives and with very high blood pressure or heart failure. The eligible residents (n=13041) were divided into newly deprescribed (reduced number of antihypertensives or dose of ≥30% after a 4-week washout) and stable users and followed up for 2 years or censored at death, discharge, or non-adherence. We used linear mixed-effects regressions to estimate per-protocol effects, with inverse probability of treatment weighting (IPTW) to adjust for confounders and additional inverse probability of censoring weighting to account for informative censoring. Analysis was stratified by dementia (yes/no) to examine the potential modification. In non-dementia subgroup (n=5954), newly deprescribed residents (n=561) had a worse ADL score (βtreat [95% CI] = 1.36 [0.82, 1.91]) compared to stable users (n=5393). Over time, ADL scores increased (worsened) 0.13 per 3 months (βtime = 0.13 [0.09, 0.17]) while antihypertensive deprescribing statistically significantly attenuated this increase by 0.25 per 3 months (βtime × treat = -0.25 [-0.48, -0.03]). The dementia subgroup showed imprecise effect (βtime × treat = -0.0003 [-0.13, 0.12]). More evidence is needed on the benefits and harms of deprescribing in long-term care populations and the importance of dementia in these relationships.

